# A Temperature-Tunable Thiophene Polymer Laser

**DOI:** 10.3390/polym10050470

**Published:** 2018-04-25

**Authors:** Mohamad S. AlSalhi, Ahlam Rashed Almotiri, Saradh Prasad, Mamduh J. Aljaafreh, Ahmad H. S. Othman, Vadivel Masilamai

**Affiliations:** 1 Research Chair on laser diagnosis of cancers, Department of Physics and Astronomy, College of Science, King Saud University, Riyadh 11451, Saudi Arabia; srajendra@ksu.edu.sa (S.P.); aouthman@ksu.edu.sa (A.H.S.O.); mvadivel@ksu.edu.sa (V.M.); 2 Department of Physics and Astronomy, College of Science, King Saud University, Riyadh 11451, Saudi Arabia; hlloom.156@gmail.com (A.R.A.); maljaafreh@ksu.edu.sa (M.J.A.)

**Keywords:** amplified spontaneous emission, temperature-tunable emission, thiophene conjugated polymer, dimer laser

## Abstract

This paper reports a temperature-tunable conjugated polymer poly[3-(2-ethyl-isocyanato-octadecanyl)-thiophene] (TCP) laser working in superradiant (SR)—or amplified spontaneous emission (ASE)—mode. The absorption spectra indicated the aggregate (mostly dimer) formation upon increasing concentration and/or decreasing temperature. Amplified spontaneous emission (ASE) was observed at suitable concentration, temperature, and pump energy values. The efficiency of the ASE from the TCP polymer was improved by energy transfer from an oligomer 9,9,9′,9′,9″,9″-hexakis(octyl)-2,7′,2′,7″-trifluorene (HOTF). Moreover, the ASE wavelength can be tuned between 550 and 610 nm by changing the temperature of the solution from 60 to 10 °C. To the best of our knowledge, this is the first report of a high-power, temperature-tunable, and conjugated polymer laser.

## 1. Introduction

The emission wavelength of a laser is critical in determining its applications. Tunable lasers are necessary for numerous applications and researches, especially in the medical, engineering, and physical sciences and in various other fields. Tunability avoids the need for different lasers for different applications. Until now, only a few optically pumped tunable lasers, such as Ti:sapphire and Cr:Forsterite, have been developed. But these solid-state lasers are very expensive and delicate to handle, and have limited tunability. Comparatively, dye lasers based on rhodamines and coumarin derivatives are cheaper and have excellent tunability, nevertheless suffer from poor photochemical stability. On the other hand, Conjugated polymers are new types of laser materials with higher photochemical stability than conventional dye solutions. Hence, optically pumped and tunable lasers based on conjugated polymers in solution represent interesting alternatives to dye lasers.

Many types of conjugated polymers are based on building blocks such as polypyrrole, polyaniline [1], polythiophene [2], and polyacetylene [3] monomers. Among them, polythiophene possesses unique electrical and optical properties as well as very good thermal stability and fluorescence properties. Polythiophene polymers find application in many fields such as organic light-emitting diodes (OLEDs) [4], solar cells [2,5,6,7], and biosensors [8].

Heavy atoms like S in any molecule were considered as florescence quenchers, despite a few molecule of oligomers being able to produce an amplified spontaneous emission (ASE) or laser.

ASE, otherwise called mirrorless or superradiant lasing, was demonstrated from thiophene molecules of 2,6-bis(5-phenylthiophen-2-yl) anthracene (BPTA), 2,6-bis(5-phenylthiophen-2-yl) naphthalene (BPTN), and 2,6-bis(5-phenylthiophen-2-yl)-1,1′-biphenyl (BPTB) single crystals when pumped with a 337 nm N_2_ laser pulse of nanosecond width [9]. It is important to emphasize at this point that any active media capable of producing ASE will produce a laser when kept in a suitable resonator cavity, however, the reverse is not true. To understand the photophysics of any active media, ASE is the most direct technique.

Optical gain and lasing in spin-coated films of a thiophene oligomer [3,3′,4′′′,3′′′′-tetracyclehexyl-3′′,4′′-dihexyl-2,2′:5′,2′′:5′′,2′′′:5′′′,2′′′′:quinquethiophene-1′′,1′′-dioxide] (T5oCx) was previously demonstrated; line narrowing in this system was obtained with a low threshold (20 µJ·cm^−2^) and the gain cross-section was 6 × 10^−16^ cm^2^, proving that a photon-emitting moiety with five sulfur atoms can still represent an active material for organic solid-state lasers [10].

Optical gain and ASE also have been obtained from thiophene oligomers by pulsed laser pump (femtosecond and nanosecond) [11,12,13,14]. A few thiophene based dyes (e.g., aza-dipyrromethene boron difluoride, Aza-BODIPY) [15] and the oligomers cited above have been reported to produce ASE, mostly in solid-state (crystal) lasers or thin films. Thiophene polymer Poly(3-(2,5-dioctylphenyl)thiophene) (PDOPT) as a thin film sandwiched between a distributed feedback (DFB) microcavity and pumped with a femtosecond (fs) pulse from Ti:Sapphire was found to produce a laser at 530 nm [16]. Here, we report ASE from a thiophene macromolecule in liquid solution. In addition, poly[3-(2-ethyl-isocyanato-octadecanyl)-thiophene] (TCP) shows a temperature dependent ASE wavelength tunability. The optical engineering and laser design using TCP has been patented by our team recently [17].

In this paper, we present a high-power and temperature-tunable laser based on the conjugated poly[3-(2-ethyl-isocyanato-octadecanyl)-thiophene] (TCP) polymer. The wavelength of this laser can be controlled in the 550–610 nm range by varying the temperature from 60 to 10 °C.

## 2. Materials and Methods

**Figure 1:**
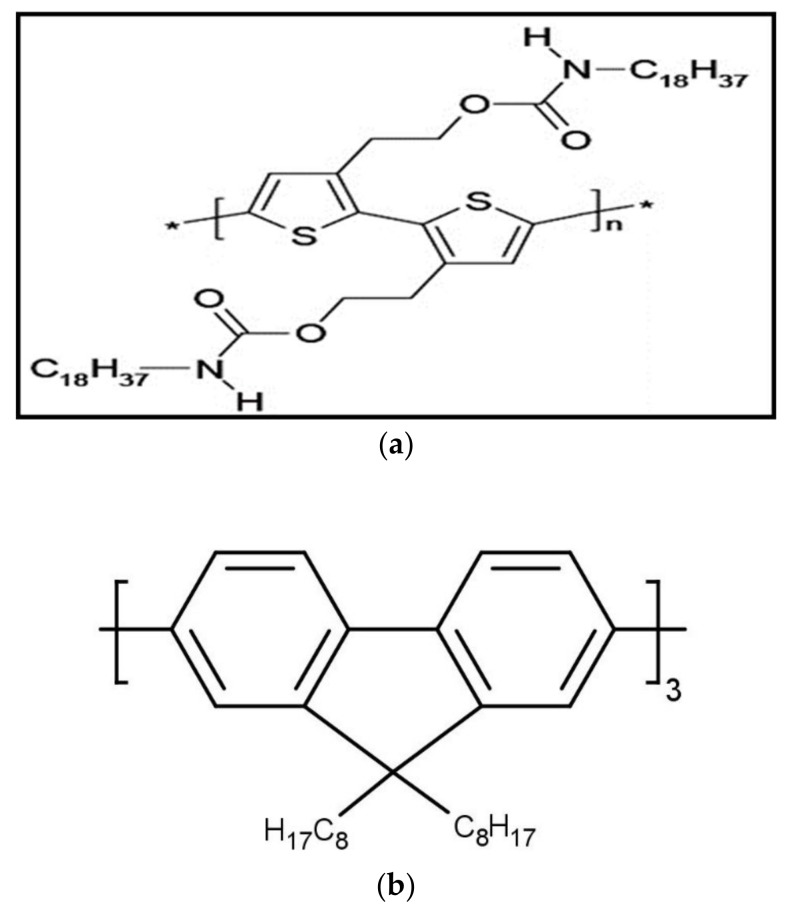
Molecular structure of (**a**) poly[3-(2-ethyl-isocyanato-octadecanyl)-thiophene] (TCP) polymer and (**b**) 9,9,9′,9′,9″,9″-hexakis(octyl)-2,7′,2′,7″-trifluorene (HOTF) oligomer.

The conjugated TCP polymer, 9,9,9′,9′,9″,9″-hexakis(octyl)-2,7′,2′,7″-trifluorene (HOTF) oligomer, and poly[2-methoxy-5-(2-ethylhexyloxy)-1,4-phenylenevinylene] (MEH-PPV) were purchased from American Dye Source (Montreal, QC, Canada) and used as received. The molecular masses of TCP, HOTF, and MEH-PPV are 190,000, 773.12, and 100,000 g·mol^−1^ respectively. The purity of the sample was estimated as >97% by thin layer chromatography (TLC). The molecular structures of polymer and oligomer are shown in Figure 1. A quartz cuvette of dimensions (1 cm × 1 cm × 4 cm) and with optical path length 10 mm was used to measure the absorption, emission, and laser properties of the TCP polymer solutions. TCP was dissolved in benzene (spectroscopic grade, 99.8% purity) in a wide range of concentrations (1 to 15 µM). The absorption and fluorescence spectra of solutions of TCP in benzene with different concentrations were recorded. Absorption spectra were recorded using a Perkin-Elmer Lambda 950 spectrophotometer (Llantrisant, United Kingdom) in the 330–550 nm range, whereas fluorescence spectra were recorded with a Perkin-Elmer LS-55 spectrofluorometer in the 380–700 nm range at room temperature and with a 355 nm excitation wavelength.

The excitation source for the ASE measurements was the third harmonic of an Nd:YAG laser [(355 nm, 10 ns, 10 Hz) (Les Ulis, France)]. A quartz plano-convex lens of 50 mm focal length was used to focus the UV laser pulse to transversely excite the liquid solution placed in a quartz cuvette. At optimum concentration and pump energy, superradiant emission, as a narrow cone of light, was observed at 550 nm. For further details, see references [18,19,20,21]. To measure the output power of ASE, we used a Maestro power energy meter (Gentec, Québec, QC, Canada).

## 3. Results and Discussion

### 3.1. Absorption and Fluorescence Properties

**Figure 2:**
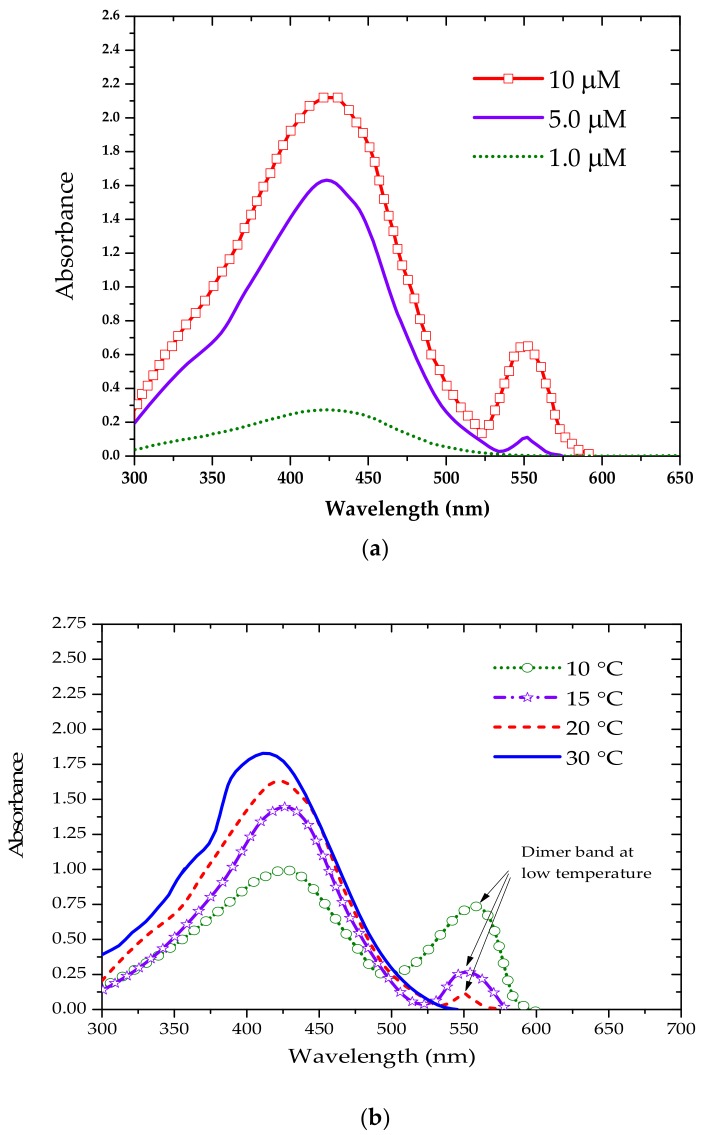
(**a**) Absorption spectra of TCP in benzene for different concentration at 20 °C; (**b**) Absorption spectra of 5 µM solution of TCP in benzene at different temperatures.

The ground-state aggregation depends strongly on the temperature, but also on the concentration. In order to evaluate the effect of the concentration on the absorption spectrum, TCP was dissolved in benzene for a range of 1 to 10 µM concentrations. At room temperature, the main peak was found around 430 nm for the 1 µM solution, whereas the 5 µM solution exhibited a main peak at 440 nm and an additional peak at 550 nm as shown in Figure 2a. The new peak at 550 nm was one third of the peak at 440 nm $\left({\mathrm{Absorbance}\;}_{\frac{550\;\mathrm{nm}}{440\;\mathrm{nm}}}=0.33\right)$ for the 10 µM concentration.

Figure 2b shows the absorption spectra of a 5 µM TCP solution in benzene at temperatures ranging from 10 to 30 °C. The main absorption peak was detected around 430 nm at 30 °C, and when the temperature was reduced to 20 °C, a new peak appeared at 550 nm. A new secondary peak at 550 nm appeared at lower temperature (<20 °C), with the main peak located at 440 nm; the peak at longer-wavelengths could be attributed to ground-state aggregations (most likely the dimer). With a further reduction in temperature to 15 °C, the peak around 550 nm became more pronounced; these spectral changes could be reproduced by changing the temperature. 

**Figure 3:**
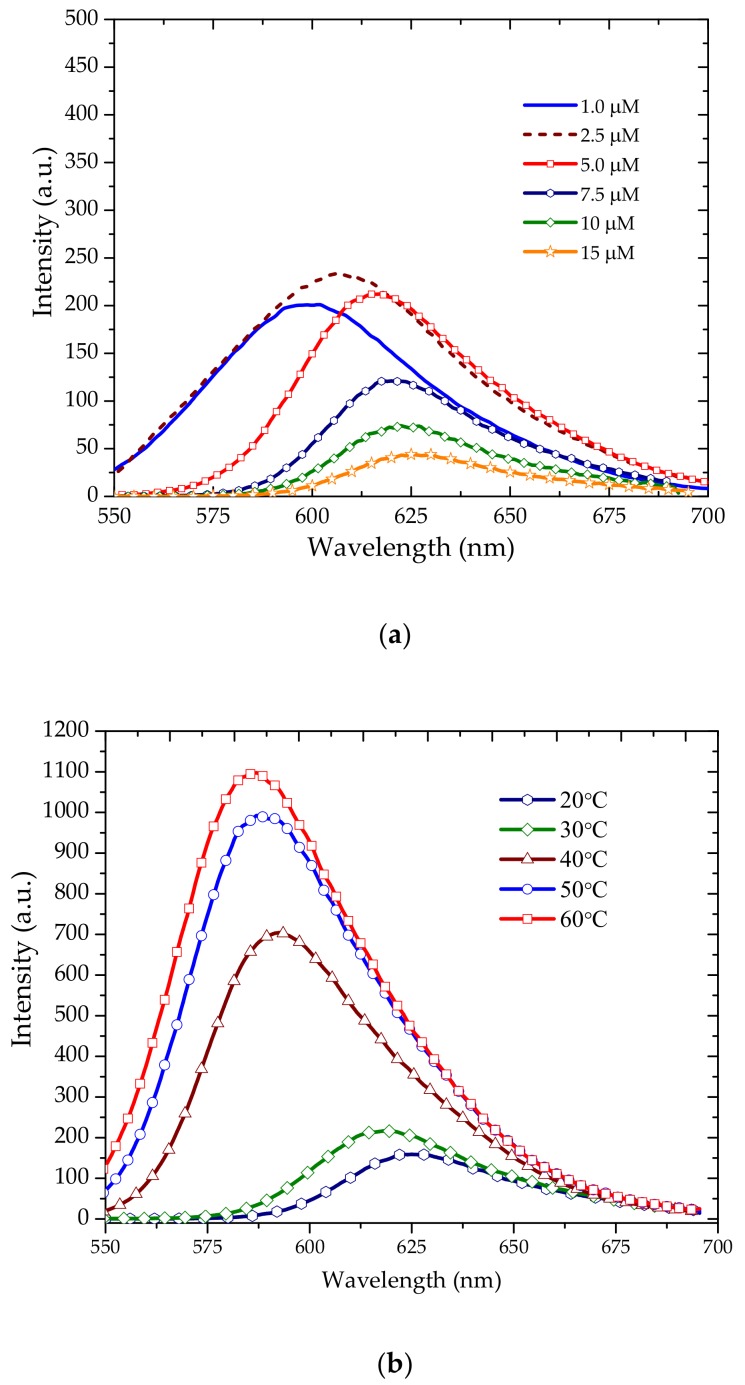
(**a**) Fluorescence spectra of TCP in benzene in different concentrations at 20 °C; (**b**) Fluorescence spectra of 15 µM TCP in benzene at different temperatures.

The Figure 3a shows the fluorescence spectra for different concentrations at 20 °C. The peak of fluorescence was at 625 nm for the 15 µM concentration; when the concentration was reduced to 7.5 µM, the peak blue shifted to 620 nm and fluorescence intensity increased. This trend continued until 2.5 µM (with peak at 605 nm), after which it fell due to dilution. The blue shift and increase in intensity of fluorescence were not as rapid as they were for the increase in temperature as shown below (Figure 3b).

Figure 3b shows the fluorescence spectra of a 5 µM solution of TCP in benzene. The fluorescence peak was found at 625 nm at 20 °C and exhibited blue shifts to 615, 590, 587, and 583 nm at temperatures of 30, 40, 50, and 60 °C, respectively.

Interestingly, the quantum yield of TCP increases with increasing solution temperature for a wide range of concentrations (further details on the method used to calculate the quantum yield can be found in reference [22]). The quantum yield (QY) was measured for different temperatures and the reference was rhodamine 6G.

$$\phi_{f}\left(s\right)=\phi_{f}\left(R\right)\;\frac{\int I_{S\;}\left(\;\bar{\upsilon }\right)\;d\left(\;\bar{\upsilon }\right)}{\int I_{R\;}\left(\;\bar{\upsilon }\right)\;d\left(\;\bar{\upsilon }\right)}\;{\left(\frac{n_{s}}{n_{R}}\right)}^{2}\left(\frac{OD_{R}}{OD_{S}}\right)$$

where *OD* is the optical density (absorbance) at the excitation wavelength (i.e., 355 nm), *n* is the refractive index of solvent, $I_{s}$, $I_{R}$ is normalized intensity, and *S*and *R* refer to sample and reference, respectively.

**Figure 4:**
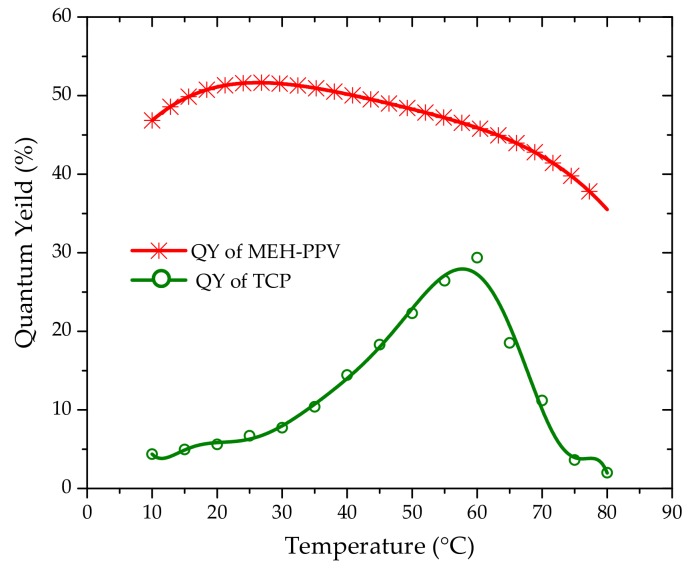
Quantum yield of poly[2-methoxy-5-(2-ethylhexyloxy)-1,4-phenylenevinylene] (MEH-PPV) of concentration 0.5 µM in benzene (red line with asterisk) and TCP of concentration 10 μM (green line with circles) at different temperatures. Note that with increasing temperature, the TCP quantum yield increases, but MEH-PPV quantum yield decreases.

The Figure 4 shows the quantum yield of TCP solution of 10 µM and at different temperatures. The QY increased with increased temperature up to 60 °C, and after that it rapidly fell. This behavior is quite rare in conjugated polymers. For example, we studied the behavior of MEH-PPV in benzene at a concentration of 0.5 µM and it was clear that quantum yield decreased with temperature. So TCP appears to exhibit an anomalous behavior. The plausible explanation for this behavior could be dissociation of aggregates at higher temperatures due to the temperature sensitivity of TCP.

### 3.2. Amplified Spontaneous Emission

When pumped by a high power laser, the inversion density and the optical gain of certain materials are so high that they simultaneously produce spontaneous and stimulated emission, resulting in a spectrally and spatially narrow cone of intense light; this process is called amplified spontaneous emission. Many thiophene polymers fail to produce ASE owing to their very low optical gain. However, the present TCP polymer shows a reasonable level of ASE.

**Figure 5:**
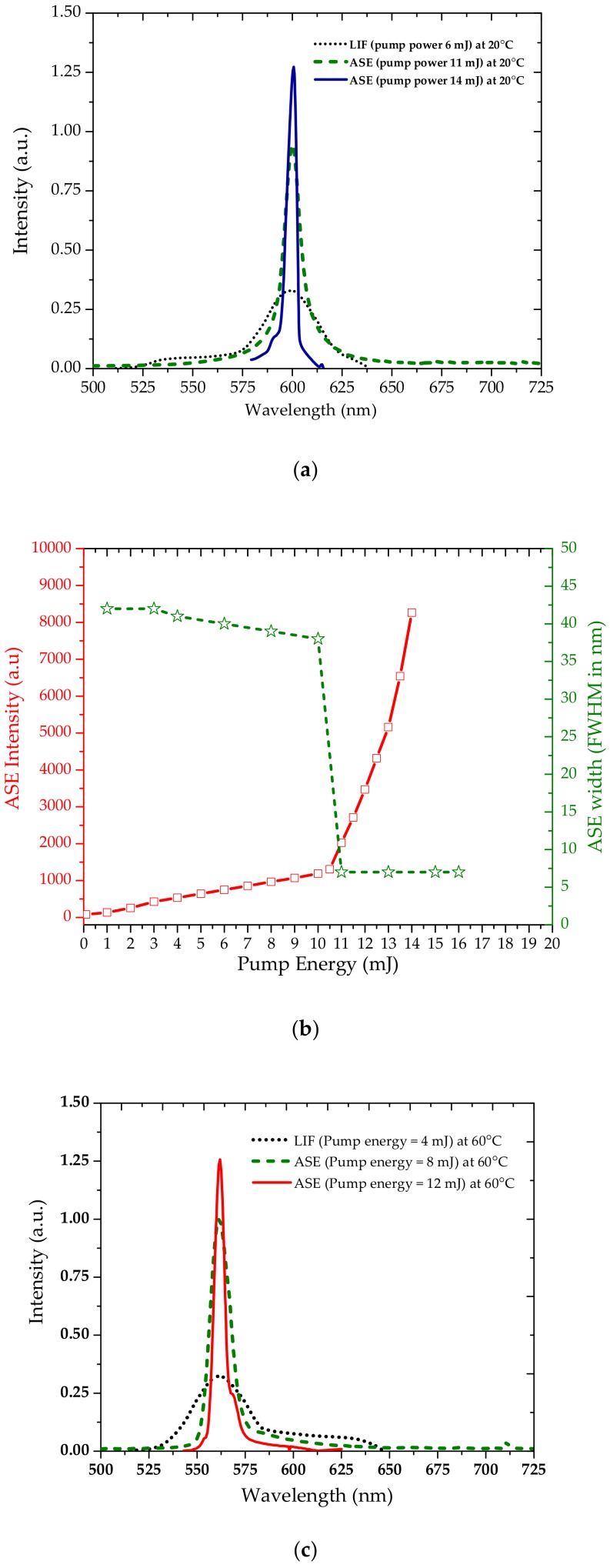

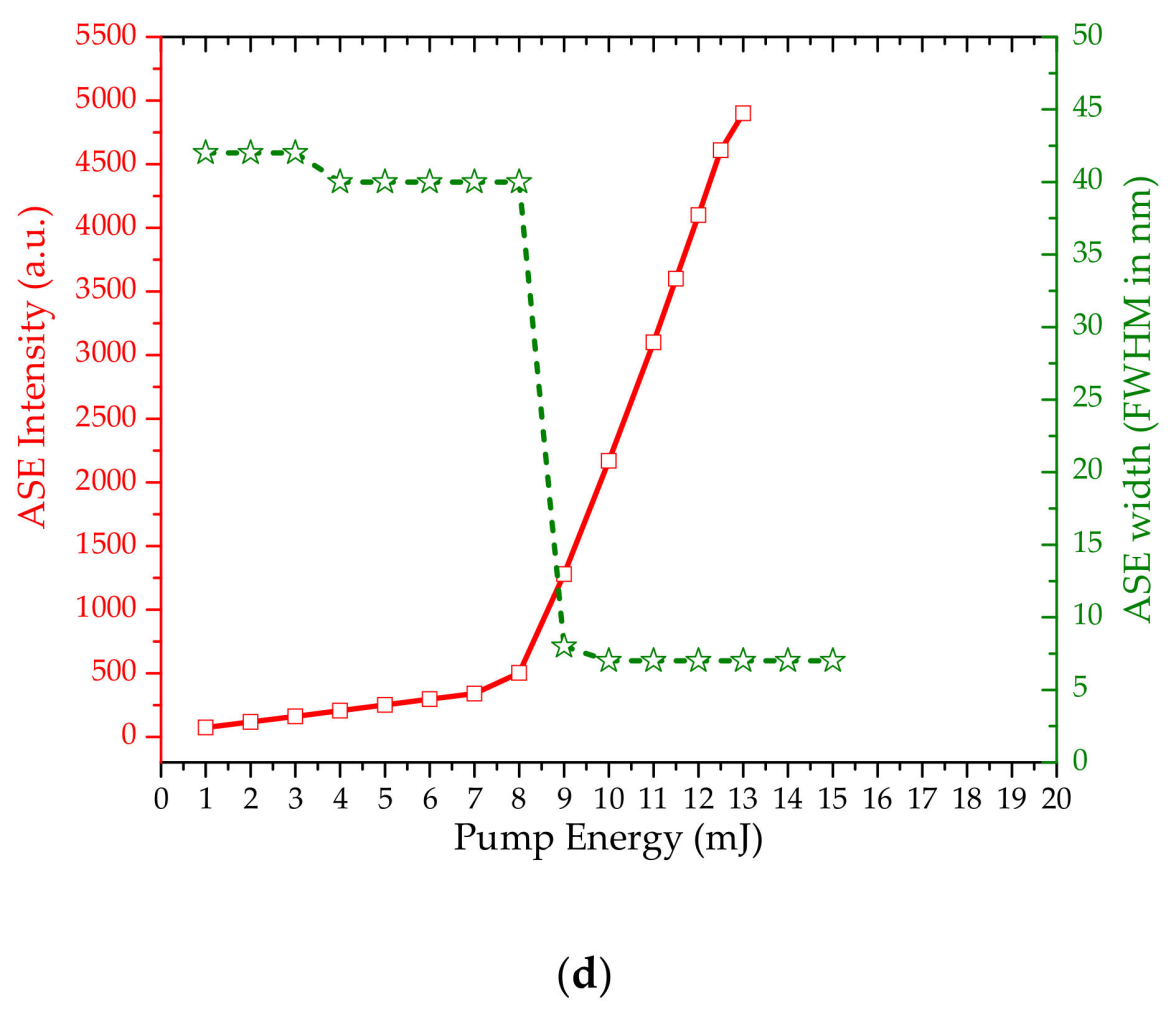
(**a**) Laser-induced fluorescence (LIF) (black dotted-line) at pump energy of 6 mJ, amplified spontaneous emission (ASE) (green dashed line) at pump energy of 11 mJ (threshold), and ASE (blue line) at pump energy of 14 mJ, for 10 µM TCP in benzene at 20 °C. (**b**) Plot between pump energy and output LIF intensity for same solution at 20 °C. (**c**) LIF (black dotted-line) at pump energy of 4 mJ, ASE (green dashed line) at pump energy of 8 mJ (threshold), and ASE (red line) at pump energy of 12 mJ for 10 µM TCP in benzene at 60 °C. (**d**) Plot between pump energy and output ASE intensity for same solution at 60 °C.

Figure 5a shows the laser-induced fluorescence (LIF) spectra of TCP for 10 µM in benzene at 20 °C, for a pump energy of 6 mJ. The main LIF peak (corresponding to the dimer) is found at 600 nm. When the pump energy was increased to 14 mJ, a distinct ASE peak at 600 nm with full width at half maximum (FWHM) of 7 nm and collimation of 10 mrad emerged. Figure 5b shows the relationship between pump energy and intensity and spectral full width half maximum (FWHM) of ASE for the same concentration and temperature (20 °C). It can be seen that 11 mJ is the threshold pump energy, after which ASE “takes off” with an intense output; consequently, there is a dramatic reduction in spectral width from 42 nm to 7 nm. Figure 5c shows that the LIF and ASE peaks shift to 550 nm, with a residual LIF peak at 600 nm, when the temperature of the solution is increased to 60 °C. Figure 5d shows the relationship between pump energy and intensity and spectral full width half maximum (FWHM) of ASE for the same concentration and temperature (60 °C). This indicates that the ASE wavelength can be varied from 600 to 560 nm by increasing the temperature from 20 to 60 °C. Figure 5d shows the relationship between pump energy and ASE intensity for the same concentration and temperature (60 °C). Note that the threshold pump energy at 60 °C was less than that at 20 °C because of the increase in quantum yield with the increase in the temperature. This behavior of TCP is quite opposite to many of the other known laser conjugated-polymers. For conventional conjugated-polymers, the threshold pump energy would increase at high temperatures because of thermal agitation and collisions.

### 3.3. Energy Transfer between Oligomer and TCP

**Figure 6:**
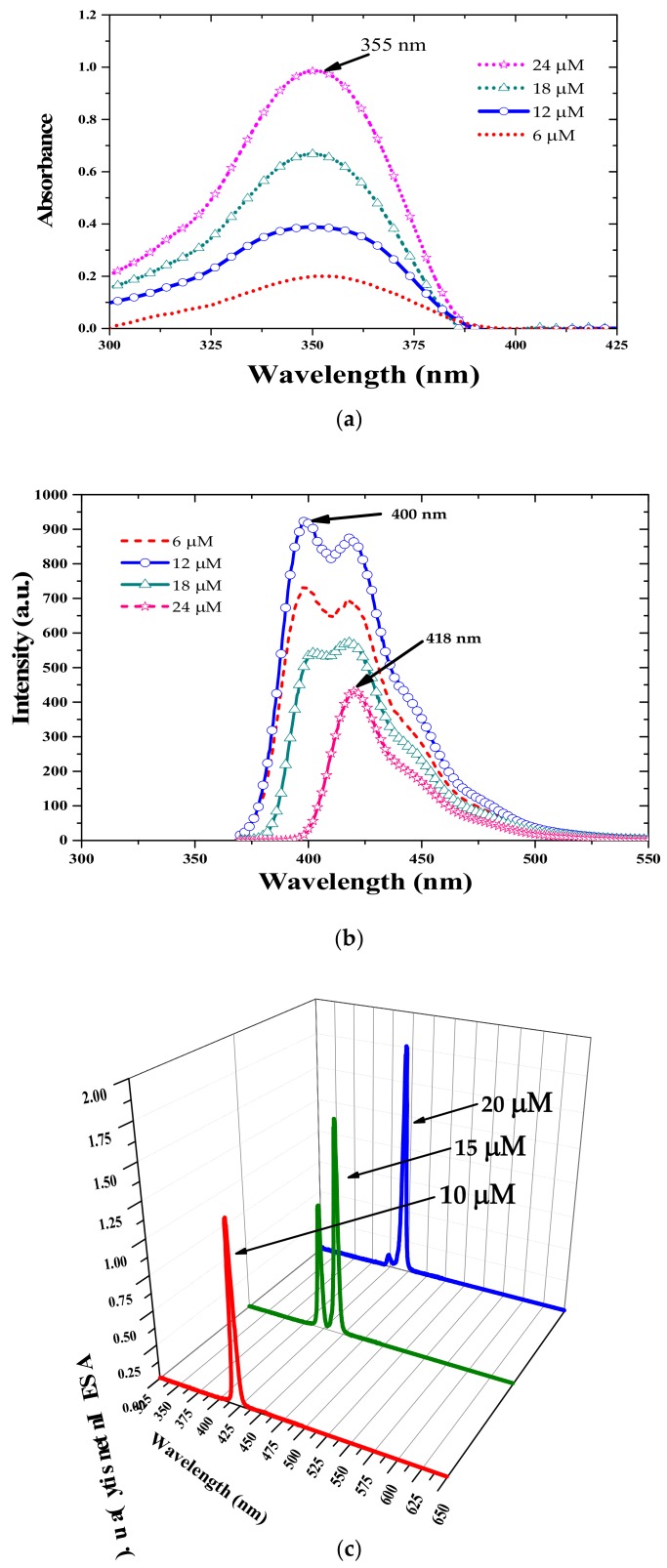
(**a**) Absorption and (**b**) fluorescence spectra of HOTF oligomer in benzene at concentrations ranging from 6 to 24 µM. (**c**) ASE forms pure oligomer in benzene at concentrations of 10, 15, and 20 μM.

In order to enhance the ASE output from the TCP, we employed an energy transfer technique. Oligomers have a small number of repeating units (3–10 monomers) and are intermediate between a monomer and a polymer. Only a few conjugated oligomers produce efficient lasers [19] and can be used as energy donors for other laser media; herein, the HOTF oligomer was employed for this purpose. Figure 6a shows the absorption spectra of HOTF solutions with concentration ranging from 6 to 24 µM. The spectral profiles of these solutions are unchanged, apart from an increase in absorbance with increasing concentration. Figure 6b shows the fluorescence spectra of the same samples, at 400 and 418 nm. The absorption and fluorescence spectra have a similar profile, with a Stokes shift of about 45 nm. Figure 6c shows the ASE from pure oligomer (HOTF) at different concentrations at 20 °C. At 10 μM, the ASE peak was at 420 nm, when the concentration was increased to 15 μM, the oligomer produced dual ASEs, one at 420 nm and the other at 440 nm. At 20 μM concentration, the peak at 420 nm almost disappeared and the 440 nm peak was dominant. The peaks at 420 and 440 nm could be attributed to the monomeric and excimeric state of the oligomer [19].

The efficiency of a Förster-type energy transfer between a donor and acceptor depends on the spectral overlap between the fluorescence of the donor and absorption of the acceptor. However, another important parameter is the Förster transfer radius (*R*_0_). When the intermolecular separation is less than or equal to this critical distance, acceptor molecules will compete well with all other decay routes. This system works well for distances of about 10 nm or less. *R*_0_ is proportional to the spectral overlap of the emission (donor) and the absorption of the acceptor and can be calculated using the following equation:

$$R_{0}^{6}=\;\frac{9000\;(\ln10)\;k^{2}\emptyset_{D}}{128\;\pi^{5}n^{4}N_{0}}\;\int \frac{F_{D}\left(\bar{\upsilon }\right)\varepsilon_{A}\left(\bar{\upsilon }\right)\;d\bar{\upsilon }}{{\bar{\upsilon }}^{4}}$$

In the above equation, *k* is the orientation factor equal to 2/3 as the medium is isotropic, $\varepsilon_{A}\left(\bar{\upsilon }\right)$ is the molar extinction coefficient of the acceptor, n is the refractive index of the solvent, $\emptyset_{D}$ is the QY of donor, $F_{D}\left(\bar{\upsilon }\right)$ is the spectral distribution of the donor normalized to unity, $\bar{\upsilon }$ is the wave number, and $N_{0}$ is Avogadro’s number. The *R*_0_ is calculated, and given below in Table 1, for a solution TCP in benzene at different temperature. For more information about the theoretical aspect of this equation, see reference [23,24].

**Figure 7:**
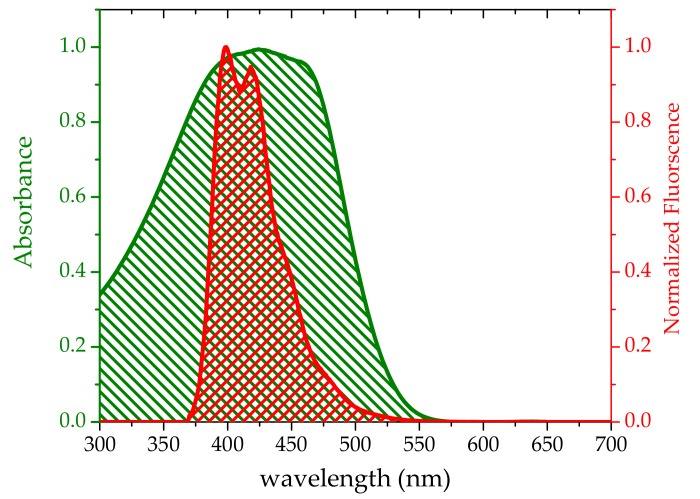
Overlap between fluorescence spectrum of HOTF (10 μM) and absorption spectrum of TCP (10 μM) at 20 °C.

Figure 7 shows that the overlap between the fluorescence spectrum of the HOTF oligomer and the absorption spectrum of TCP, indicating that the oligomer could represent a good energy donor for the TCP acceptor. We observed efficient energy transfer because of this overlap, the small value of *R*_0_ (averaging 3.46 nm), and high QY of the donor.

**Figure 8:**
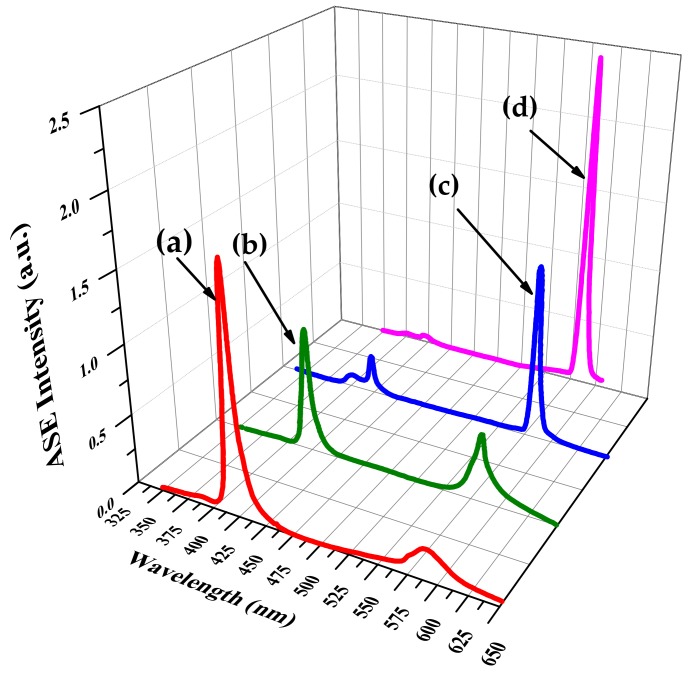
Energy transfer from oligomer to TCP; the concentration of donor and acceptor was (**a**) 9 µM/0.72 µM, (**b**) 8 µM/1.6 µM, (**c**) 7.6 µM/1.8 µM, and (**d**) 6.7 µM/2.7 µM at 20 °C.

Figure 8 shows the kinetic evolution of the energy transfer between oligomer and TCP; the effective concentration of acceptor and donor in mixed solution varied as the acceptor was added drop by drop. Without TCP, a strong ASE was observed at 420 nm from the oligomer. After placing 2 mL of oligomer in a cuvette, concentrated TCP was added dropwise (in 0.05 mL drops). Each TCP addition led to a drop in the ASE intensity of the oligomer and a corresponding dramatic increase in fluorescence of TCP. ASE was produced from the TCP when its concentration was 2.7 µM and that of the oligomer was 6.7 µM. An increase of 80% in the ASE efficiency (compared with pure TCP of a concentration 10 μM at 20 °C (Figure 5a) was achieved by determining the optimal composition of the working solution by trial and error.

**Figure 9:**
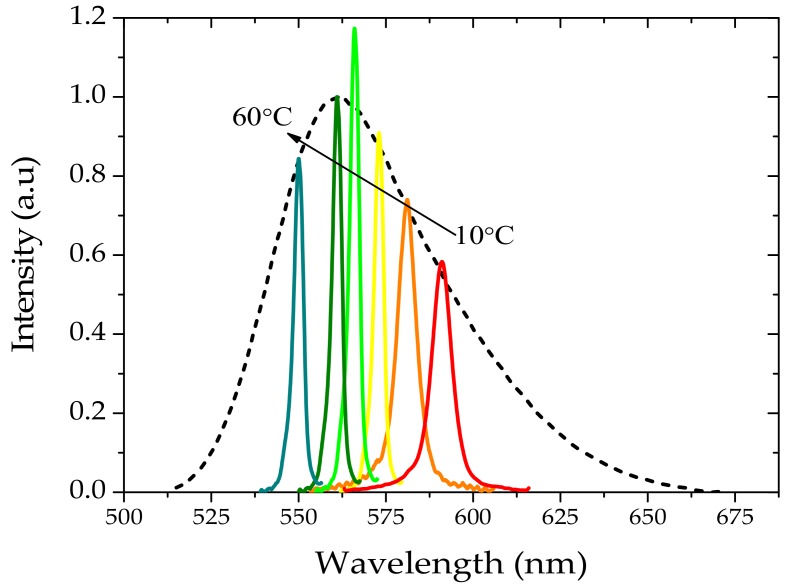
ASE from mixture of 8 µM TCP and 6 µM oligomer in benzene solution. Solid lines and dotted lines are ASE spectra with and without energy transfer from oligomer. The energy transfer increases the ASE intensity.

Whereas a reasonable ASE output could be achieved with different donor (oligomer)/acceptor (TCP) combinations, the best results were obtained by mixing 8 µM oligomer and 2 µM TCP in a 1:1 volume ratio. With the pump energy fixed at 12 mJ, varying the temperature from 10 to 60 °C allowed tuning the ASE from 605 to 550 nm as shown in Figure 9. It is important to note that temperature tuning was highly reproducible and linear.

## 4. Conclusions

We report the optical design of a temperature-tunable laser based on a conjugated thiophene polymer. An increase of 80% in the ASE efficiency was achieved by employing an oligomer as energy donor. The most interesting feature of the new laser is the fully reversible temperature tunability of its wavelength in the 550–600 nm range, at a rate of 1.2 nm/°C.

## Figures and Tables

**Table 1:** The calculated values of quantum yield ($QY_{d}$) and Förster transfer radius (*R*_0_) for the TCP solution of concentration 10 μM.

Temperature	$QY_{d}$	*R*_0_ (nm)
10	0.64	3.61
20	0.62	3.63
30	0.55	3.55
40	0.50	3.52
50	0.48	3.31
60	0.44	3.18
